# Toothpick inside the Common Bile Duct: A Case Report and Literature Review

**DOI:** 10.1155/2017/5846290

**Published:** 2017-03-05

**Authors:** V. O. Brunaldi, M. O. Brunaldi, R. Masagao, C. Silva, H. Masuda, J. E. Brunaldi

**Affiliations:** ^1^Gastroenterology Department, Faculty of Medicine, University of São Paulo, 05403-001 São Paulo, SP, Brazil; ^2^Pathology Department, Faculty of Medicine of Ribeirão Preto, University of São Paulo, Ribeirão Preto, SP, Brazil; ^3^Division of Digestive Endoscopy and General Surgery, Carlos Fernando Malzoni Hospital, Matão, SP, Brazil; ^4^Digestive Surgery Department, Faculty of Medicine of Ribeirão Preto, University of São Paulo, Ribeirão Preto, SP, Brazil

## Abstract

The incidence and prevalence of foreign body (FB) ingestion are difficult to estimate. Unlike other foreign bodies, the ingestion of a toothpick is very uncommon and carries high morbidity and mortality rates. We report a case of a 73-year-old female patient presenting mid-term epigastric pain. Abdominal ultrasound revealed a slightly dilated common bile duct (CBD) and magnetic resonance showed an irregular filling failure in distal CBD and gallstones. Endoscopic Retrograde Cholangiopancreatography revealed major papilla on the edge of a diverticulum and confirmed the distal filling failure. After sphincterotomy, a partially intact toothpick was extracted from the CBD. Neither fistulas nor perforation signs were found. Literature related to foreign bodies and toothpick ingestion was reviewed and some hypotheses to explain the reported case were created. To our knowledge, this is the first report of a toothpick lodged inside the biliary tract.

## 1. Introduction

The incidence and prevalence of foreign body (FB) ingestion are difficult to estimate [1]. It is responsible for around 1.500 deaths annually in the USA [2], although mortality is proportionally very low [3]. The extremely low morbidity and mortality rates are mostly because about 80% of the ingested FBs go through the digestive tract without any major complication [2–5].

FB ingestion may lead to a wide variety of complications. To summarize, they can be divided into two major groups: obstruction-related complications and those related to overpressure in a specific point of the bowel, leading to ulceration, perforation, and fistulas.

Concerning the specific ingestion of toothpicks, more than half of all cases go unnoticed by patients and frequently lead to perforations [6]. In medical literature, there are several case reports of toothpicks lodged in a wide range of sites [6–9]. However, this is the first case report of a toothpick inside the bile duct.

## 2. Case Report

We report a case of a 73-year-old white female patient with complaints of mild epigastric pain over the last month associated with postprandial fullness. Patient denied nausea and gastrointestinal bleeding. She had no relevant past medical history. The physical exam revealed normal general appearance and the abdominal examination showed no signs of abnormal conditions.

**Figure 1:**
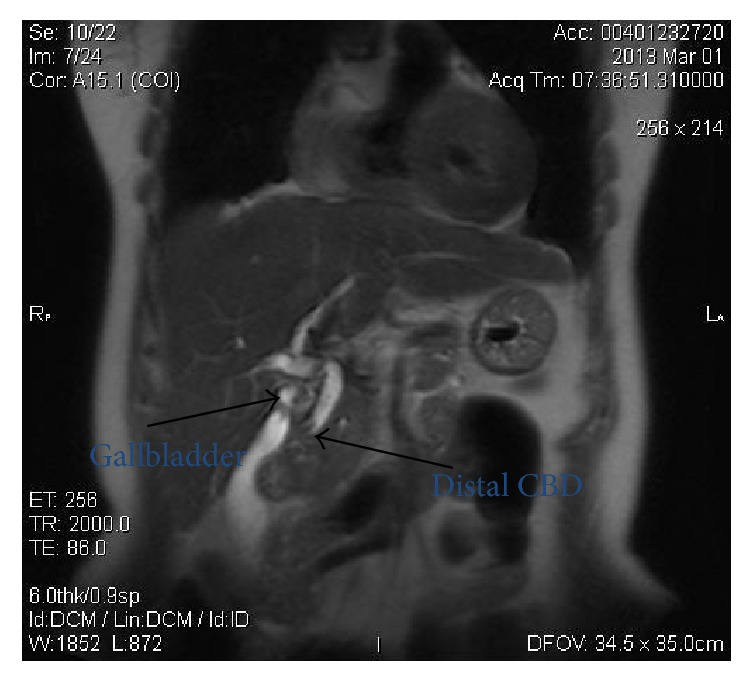
MRI: coronal section shows gallstones and the pointed filling failure in the distal common bile duct.

Workup proceeded with an abdominal ultrasound that showed a slightly dilated common bile duct (CBD) and lab results revealed normal bilirubin, amylase, and transaminases. Complementary magnetic resonance showed an irregular filling failure in the CBD and gallstones (Figure 1). Hence, the presumed diagnosis was choledocholithiasis and the patient was referred to our endoscopy department to undergo endoscopic stone extraction through Endoscopic Retrograde Cholangiopancreatography (ERCP).

**Figure 2:**
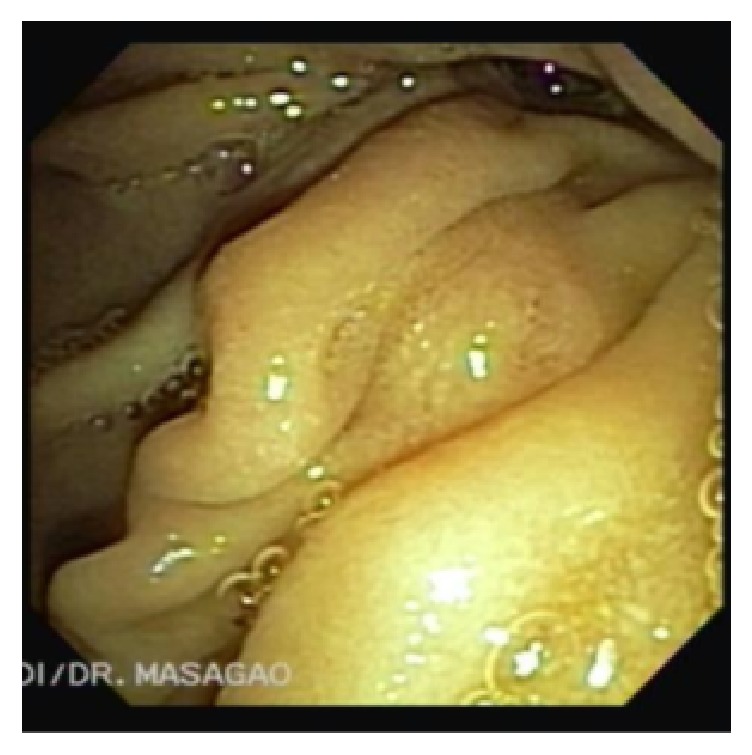
Duodenoscopy shows topic major papilla on the edge of a diverticulum filled with food bezoar (right upper quadrant).

**Figure 3:**
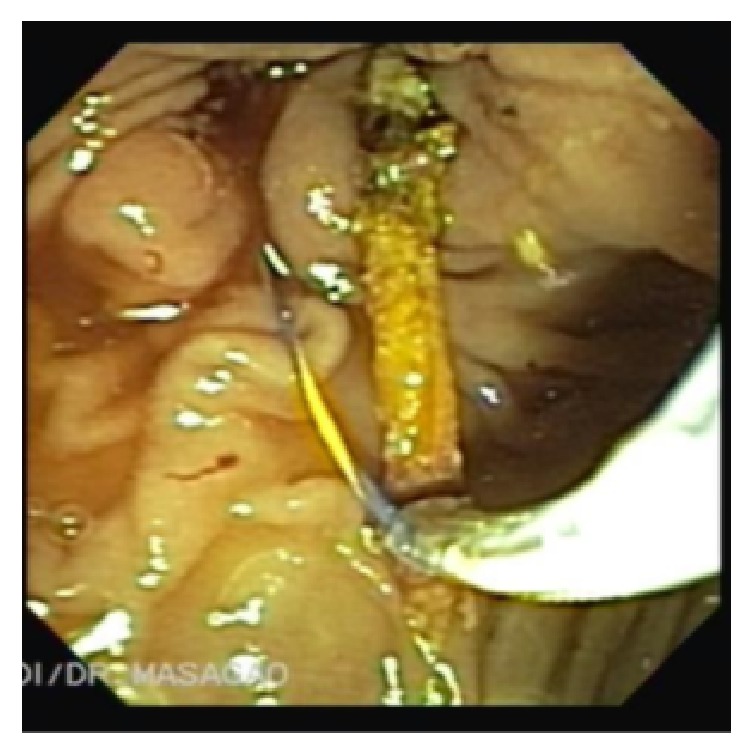
Extraction of a partially intact toothpick with a trapezoid basket.

**Figure 4:**
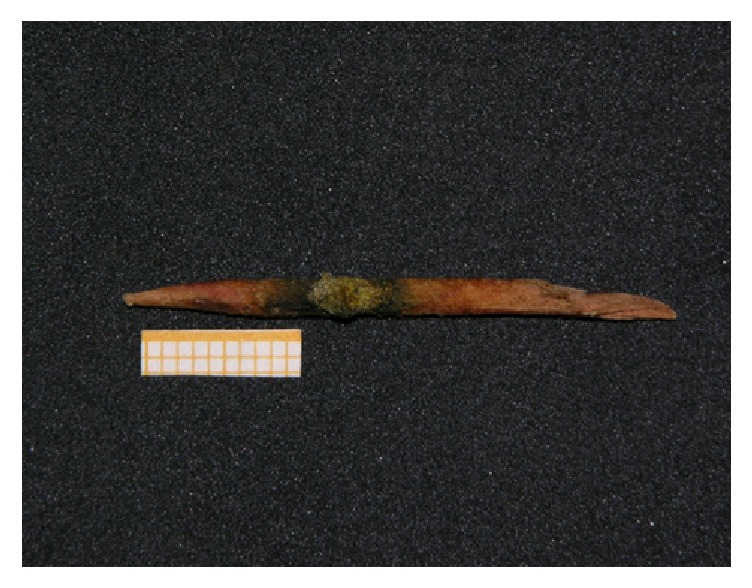
Extracted toothpick.

Duodenoscopy found the major papilla on the edge of an anterior diverticulum (Figure 2) and retrograde cholangiography confirmed the irregular filling failure at the distal CBD. After sphincterotomy, extraction of a partially intact toothpick (Figures 3 and 4) was successfully accomplished. Neither perforations nor fistulas were found.

Patient had excellent recovery and was discharged one day after the procedure. Cholecystectomy was performed after convalescence and the patient remains asymptomatic after 2-year follow-up.

## 3. Discussion

The pediatric population is the major victim of foreign body (FB) ingestion, especially patients between 6 months and the age of 6 [2, 3, 10–12]. Regarding adults, it is related to psychiatric disorders, alcoholism, neurodevelopmental delay, and intentional swallowing for smuggling purposes [3, 4, 13]. Among the elderly, it is mainly linked to improper use of dental prosthesis [14]. Also, the sort of object swallowed depends on the age and cognitive status of the patient: infants usually take small and easy-to-get objects, and adults swallow bones and other food related items, while the elderly and neurologically impaired patients usually swallow dentures [3, 4].

FB ingestion has extremely low morbidity and mortality rates, especially after the object reaches the stomach [2–5]. The size, shape, or multiplicity is not useful to predict if a FB would pass [15]. However, there are several complications and one of the most severe is perforation [16]. The major risk factors for perforation are ingestion of sharp or pointed FBs, length of stay in the digestive tract longer than 24 hours and previous gut malformation or abdominal surgery [6, 17–19]. The incidence of FB ingestion requiring surgery varies from less than 1% to 14% [4, 15, 20].

With regard to therapeutic strategy, flexible endoscopy is the gold-standard method for noncomplicated cases. It presents a success rate of around 99% and extremely low morbidity [20]. Endoscopic procedure should be preceded by adequate radiological workup which allows the correct therapeutic planning (sided view versus front view; e.g., need of endoscopic ultrasound or fluoroscopy) [1]. However, if the object reaches the stomach, asymptomatic patients can be safely observed for development of symptoms as more than 80% pass spontaneously [15].

On the contrary, toothpick ingestion poses the greatest risk of perforation [15]. Regarding specific ingestion of toothpicks, a recent literature review analyzed 136 reported cases from 1927 to 2012. More than half of all cases go unnoticed by patients (54%) and lead to perforations in almost 80% of all cases. Based on the review, an algorithm for the management of toothpick ingestion was developed by Steinbach et al. The most sensible exam for diagnosis is flexible endoscopy that presents no reported mortality when the toothpick is successfully extracted, and therefore is the first diagnostic step. Abdominal ultrasound should be the next step if gastroscopy do not detect the object or if the time interval after ingestion is longer than 24 hours. If ultrasound does not provide a definitive diagnosis, the next step should be determined by patient's clinical condition: if stable and oligosymptomatic, a conventional X-ray to exclude free gas; if unstable or signs of peritonitis, contrast-enhanced CT scan is the next step followed by urgent surgical removal. If patient is stable and the toothpick is found in colon, it should be removed by colonoscopy. After all steps, stable patients should be admitted for observation if location is not possible [6].

Perforations caused by swallowed foreign bodies at the duodenum are particularly interesting once it may not cause peritonitis but migration to adjacent organs such as pancreas, liver, and retroperitoneum [6]. Some reports describe hepatic abscesses caused by FBs. Usually, the object is metallic and sharp, but there are more than 15 case reports of toothpick migration to the liver leading to hepatic abscesses [21]. Surgical treatment for such disorder is mandatory [21]. Differently, pancreatic migration of swallowed toothpick is much less common. Some reports describe complications such as pancreatitis, pancreatic hemorrhage and pancreatic pseudotumor [1, 22]. Migration to retroperitoneum is even rarer. Right psoas muscle abscess has already been reported as a complication of duodenal perforation [23].

There are some reports of FBs inside the biliary tract. Most cases lead to biliary obstruction and the FB is usually related to past surgical or endoscopic procedures. Endoclips, suture material, and stents in cholecystectomized patients are the most common objects [24–28]. Diagnoses are usually established after presentation of obstructive jaundice due to bile duct stone formation around the FB that works as a nidus [24–26].

However, there is a different group of reported cases that are not related to previous interventional procedures. Oligosymptomatic patients presenting FBs inside the CBD without signs of perforation or fistulas. Metal pin, tomato peel (food bezoar), and fish-bones have already been reported [29–31]. A case of recurrent choledocholithiasis due to foreign body after endoscopic sphincterotomy has also been reported [32]. Whenever a choledochoenteral fistula is found, it is postulated as the route for migration of the FB. Otherwise, the most pointed mechanism is reflux from duodenum [29, 33]. Procházka et al. analyzed 54 gallstones obtained endoscopically and found foreign material in stones of 6 individuals of which 4 had previously undergone cholecystectomy. In those four patients, surgical suturing material was found. The remaining two patients presented fiber and cellulose in the gallstone [33]. Moreover, Henderson et al. performed manometry of the greater papilla in patients with choledocholithiasis due to FBs and compared to manometry of patients with common stones. Patients with stones due to FBs presented greater prevalence of retrograde waves compared to patients with typical stones [16]. These studies support duodenal reflux as a theory.

Concerning the diverticulum, duodenum is the second most common location after colon. They are usually asymptomatic acquired disorders. Only 1 to 5% become symptomatic and symptoms are usually related to complications such as gastrointestinal, biliary or pancreatic obstruction, perforation and hemorrhage. Endoscopic treatment is the gold-standard for biliopancreatic complications and bleeding [34]. Moreover, the juxtapapillary diverticulum is strongly associated with choledocholithiasis [35]. Manometric studies of the sphincter of Oddi in patients presenting juxtapapillary diverticulum found that muscular tone, contractile activity, and total rhythmic variation are significantly less compared to patients without diverticulum [36]. This dysfunction of the sphincter of Oddi may lead to bile stasis and duodenal reflux.

In our case, we hypothesize that the duodenal diverticulum has fundamental role since it propitiates food stasis and provides an adequate location for the toothpick to twine on. Also, it probably led to dysfunction of the sphincter of Oddi and to greater duodenal reflux. The reflux allowed retrograde flow of the toothpick into CBD. The combination of such rare and independent risk factors makes this report unique.

## 4. Conclusion

Different from other FBs, toothpick ingestion is a rare disorder and demands specific medical care. Given its rare nature, case reports and case series are very important tools to correctly understand and treat victims. Besides, the presented clinical case may exemplify another physiopathology for primary choledocholithiasis: duodenal reflux into biliary tract. More studies are certainly needed to endorse our hypothesis.
